# Standards of diagnostic colonoscopy for early‐stage neoplasia: Recommendations by an Asian private group

**DOI:** 10.1111/den.13330

**Published:** 2019-03-29

**Authors:** Yasushi Sano, Han‐Mo Chiu, Xiao‐bo Li, Supakij Khomvilai, Pises Pisespongsa, Jonard Tan Co, Takuji Kawamura, Nozomu Kobayashi, Shinji Tanaka, David G. Hewett, Yoji Takeuchi, Kenichiro Imai, Takahiro Utsumi, Akira Teramoto, Daizen Hirata, Mineo Iwatate, Rajvinder Singh, Siew C. Ng, Shiaw‐Hooi Ho, Philip Chiu, Hisao Tajiri

**Affiliations:** ^1^ Gastrointestinal Center and Institute of Minimally invasive Endoscopic Care (iMEC) Sano Hospital Hyogo; ^2^ Department of Gastroenterology Kyoto Second Red Cross Hospital Kyoto Japan; ^3^ Department of Gastroenterology and Hepatology Kyoto University Graduate School of Medicine Kyoto Japan; ^4^ Department of Gastroenterology Tochigi Cancer Center Tochigi Japan; ^5^ Department of Endoscopy Hiroshima University Hospital Hiroshima Japan; ^6^ Department of Gastrointestinal Oncology Osaka International Cancer Institute Osaka Japan; ^7^ Division of Endoscopy Shizuoka Cancer Center Shizuoka Japan; ^8^ Department of Innovative Interventional Endoscopy Research The Jikei University School of Medicine Tokyo Japan; ^9^ Department of Internal Medicine College of Medicine National Taiwan University Taipei Taiwan; ^10^ Division of Gastroenterology and Hepatology Key Laboratory of Gastroenterology and Hepatology Ministry of Health Renji Hospital School of Medicine Shanghai Institute of Digestive Disease Shanghai Jiao Tong University Shanghai China; ^11^ Departments of Medicine and Therapeutics Institute of Digestive Disease State Key Laboratory of Digestive Diseases LKS Institute of Health Science The Chinese University of Hong Kong Hong Kong China; ^12^ Surgery Institute of Digestive Disease State Key Laboratory of Digestive Diseases LKS Institute of Health Science The Chinese University of Hong Kong Hong Kong China; ^13^ Surgical Endoscopy Colorectal Surgery Department of Surgery Chulalongkorn University Bangkok Thailand; ^14^ Digestive Disease Center Bumrungrad International Hospital Bangkok Thailand; ^15^ St. Luke's Medical Centre ‐ Global City Taguig City, Metro Manila Philippines; ^16^ Faculty of Medicine University of Queensland Brisbane Australia; ^17^ Gastroenterology Unit Division of Medicine Lyell McEwin Hospital School of Medicine The University of Adelaide Adelaide Australia; ^18^ Department of Medicine Faculty of Medicine University of Malaya Kuala Lumpur Malaysia

## Abstract

**Background and Aim:**

In recent years, the incidence of colorectal cancer has been increasing, and it is now becoming the major cause of cancer death in Asian countries. The aim of the present study was to develop Asian expert‐based consensus to standardize the preparation, detection and characterization for the diagnosis of early‐stage colorectal neoplasia.

**Methods:**

A professional group was formed by 36 experts of the Asian Novel Bio‐Imaging and Intervention Group (ANBI^2^G) members. Representatives from 12 Asia–Pacific countries participated in the meeting. The group organized three consensus meetings focusing on diagnostic endoscopy for gastrointestinal neoplasia. The Delphi method was used to develop the consensus statements.

**Results:**

Through the three consensus meetings with debating, reviewing the literature and regional data, a consensus was reached at third meeting in 2016. The consensus was reached on a total of 10 statements. Summary of statements is as follows: (i) Adequate bowel preparation for high‐quality colonoscopy; (ii) Antispasmodic agents for lesion detection; (iii) Image‐enhanced endoscopy (IEE) for polyp detection; (iv) Adenoma detection rate for quality indicators; (v) Good documentation of colonoscopy findings; (vi) Complication rates; (vii) Cecal intubation rate; (viii) Cap‐assisted colonoscopy (CAC) for polyp detection; (ix) Macroscopic classification using indigocarmine spray for characterization of colorectal lesions; and (x) IEE and/or magnifying endoscopy for prediction of histology.

**Conclusion:**

This consensus provides guidance for carrying out endoscopic diagnosis and characterization for early‐stage colorectal neoplasia based on the evidence. This will enhance the quality of endoscopic diagnosis and improve detection of early‐stage colorectal neoplasia.

## Introduction

In recent years, the incidence of colorectal cancer (CRC) has been increasing, and it is now becoming the major cause of cancer death in Asian countries. Fecal immunochemical test (FIT) is widely used for CRC screening, and its effectiveness in reducing CRC incidence and mortality has been shown in Asia.^1^, ^2^, ^3^ Several studies have also recently indicated the effectiveness of screening colonoscopy for reducing CRC incidence and mortality.^4^, ^5^, ^6^ Although CRC screening colonoscopy is not adopted as a primary population‐based screening tool, the number of colonoscopies for screening objectives after FIT is increasing in Asia.^1^

When a patient undergoes colonoscopy, the most important task for colonoscopists is not to miss or overlook any colorectal neoplasia, especially early‐stage CRC. Early‐stage CRC is defined as cancer that is confined to the mucosa or submucosa and does not invade the muscularis propria. Intramucosal cancer is virtually never associated with lymph node metastasis and can be curatively resected by colonoscopy. Once the submucosal layer is invaded, lymph node metastasis occurs in 6–13% of cases.^7^ However, shallow submucosal invasion (SM1 or T1a), especially invasion with a vertical depth of <1000 μm from the lower border of the muscularis mucosae, can still be treated endoscopically, depending on its lateral size, endoscopic features, and histopathological features.^8^, ^9^

Colonoscopy plays a fundamental role in the prevention and management of CRC and is used for both diagnosis and treatment of early CRC and its precursor lesions. Improvements in colonoscopy preparation, documentation, new techniques of adenoma detection, and recent progress in endoscopic imaging methods are providing higher quality results and reducing the incidence and mortality of the disease.^10^, ^11^

Against this background, the Asian Novel Bio‐Imaging and Intervention Group (ANBI^2^G), which is an academic interest group, was established as a non‐governmental organization (NGO) in Hong Kong in February 2014 (http://www.anbig.org/). Consisting of world‐class health‐care professionals (HCP) in Asia, its objectives are to: (i) promote training and education in early gastrointestinal (GI) cancer diagnosis and therapy in the Asia–Pacific region; (ii) develop strategies and action plans for the professional development of endoscopists in the Asia–Pacific region; and (iii) work with any society, association or person for the purpose of furthering the education efforts of the society.

Contrary to this trend, in Asian countries, there is no consensus on diagnostic colonoscopy to recognize and characterize early‐stage colorectal neoplasia. Therefore, ANBI^2^G members have held meetings over the past 2 years to refine the consensus for diagnostic endoscopy in Asia. The aim of the present study was to develop Asian expert‐based consensus to standardize the preparation, detection and characterization for diagnosis of early‐stage colorectal neoplasia.

## Methods

A professional group was formed by 36 expert ANBI^2^G members in 2016. Representatives from 12 Asia–Pacific countries participated in the meeting: these included Australia, China, India, Indonesia, Japan, Malaysia, Myanmar, Philippines, Singapore, Korea, Taiwan, Thailand, and Vietnam. The group organized three consensus meetings focusing on diagnostic endoscopy for GI neoplasia.

### First consensus meeting (22–24 January, 2016, ANA Crowne Plaza Hotel, Kobe, Japan)

The first face‐to‐face meeting focused on drafting the consensus statements on standards of diagnostic lower GI endoscopy according to the following areas:

1. Standard preparation for diagnostic endoscopy.
2. Endoscopic recognition of early GI neoplasia.
3. Endoscopic characterization of early GI neoplasia.

An initial draft of 10 statements was formulated, discussed and agreed by the panel of experts. Each member was assigned to search for evidence of the respective statement and then prepare for discussion and voting in the following consensus meetings.

The literature search for each statement was based on publications in English from various scientific databases including AMED, BIOSIS previews, EBM reviews, Embase, Ovid MEDLINE, Cochrane Trials and systematic reviews.

### PICO method for establishment of statements

**Figure 1:**
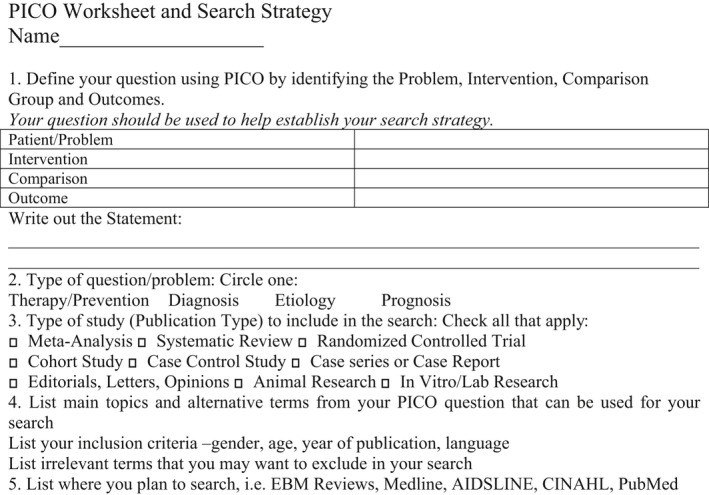
Problem/Population; Intervention; Comparison and Outcome (PICO) Worksheet and Search Strategy.

The Problem/Population; Intervention; Comparison and Outcome method (PICO) was used to identify the appropriate interventions and standards for diagnostic endoscopy focusing on detection and characterization of early GI neoplasia as a clinical outcome. All statements were established following the PICO worksheet (Fig. 1).

### Delphi method for voting

**Figure 2:**
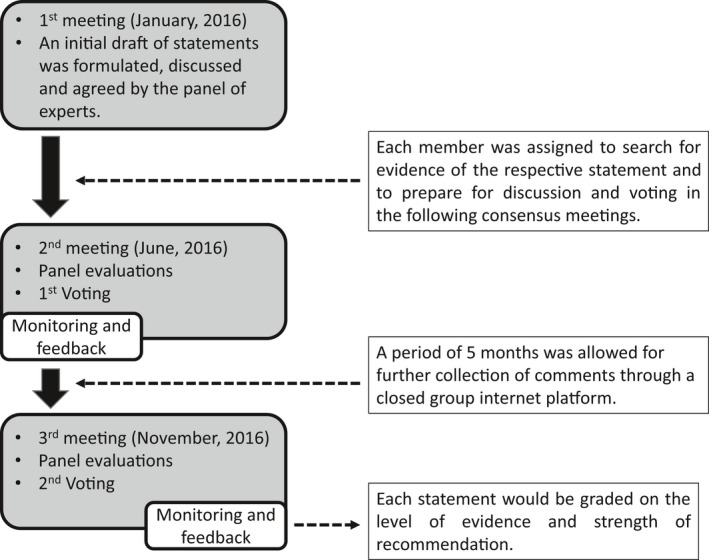
Delphi method.

The expert panel formulated statements in two separate rounds of voting and opinion collection (Fig. 2). After each round, facilitators provided summaries of expert opinions from the previous round for refinement. If consensus was not achieved, the process was continued through discussion to work towards building one. The voting was based on reviews of the literature on a Likert scale ranging from 1 to 5 (1 = accept completely, 2 = accept with some reservation, 3 = accept with major reservation, 4 = reject with reservation, 5 = reject completely). All voting was held at the end of the talks using a wireless polling system to ensure anonymity.

Consensus was achieved when >80% of members indicated that they accepted the statement completely or accepted it with some reservation. When consensus was not reached at the first vote, panel members discussed the statement again for further modification. This was followed by a second round of voting. If there was still failure to reach a consensus, the statement was rejected. Each statement was graded on the level of evidence and strength of recommendation (Tables 1 and 2).

**Table 1:** Quality of evidence summarized for each of the statements will be graded according to the classification below

	Level of evidence
I	Evidence obtained from at least one randomized controlled trial
II‐1	Evidence obtained from well‐designed control trials without randomization
II‐2	Evidence obtained from a well‐designed cohort or case–control study
II‐3	Evidence obtained from comparison between time or places with or without intervention
III	Opinion of respected authorities, based on clinical experience and expert committees

**Table 2:** Classification of recommendations

	Grade of recommendation
A	There is good evidence to support the statement
B	There is fair evidence to support the statement
C	There is poor evidence to support the statement but recommendation made on other grounds
D	There is fair evidence to refute the statement
E	There is good evidence to refute the statement

### Second consensus meeting (23–24 June 2016, Swissotel Nankai, Osaka, Japan)

After the search for evidence on 10 statements, a second face‐to‐face meeting was conducted in order to vote on and refine the statements. An appropriate time was allowed for each statement through discussion among members to allow for refinement. After the meeting, a period of 5 months was allowed for further collection of comments through a closed group internet platform.

### Third consensus meeting (6 November 2016, Sano Hospital, Kobe, Japan)

**Figure 3:**
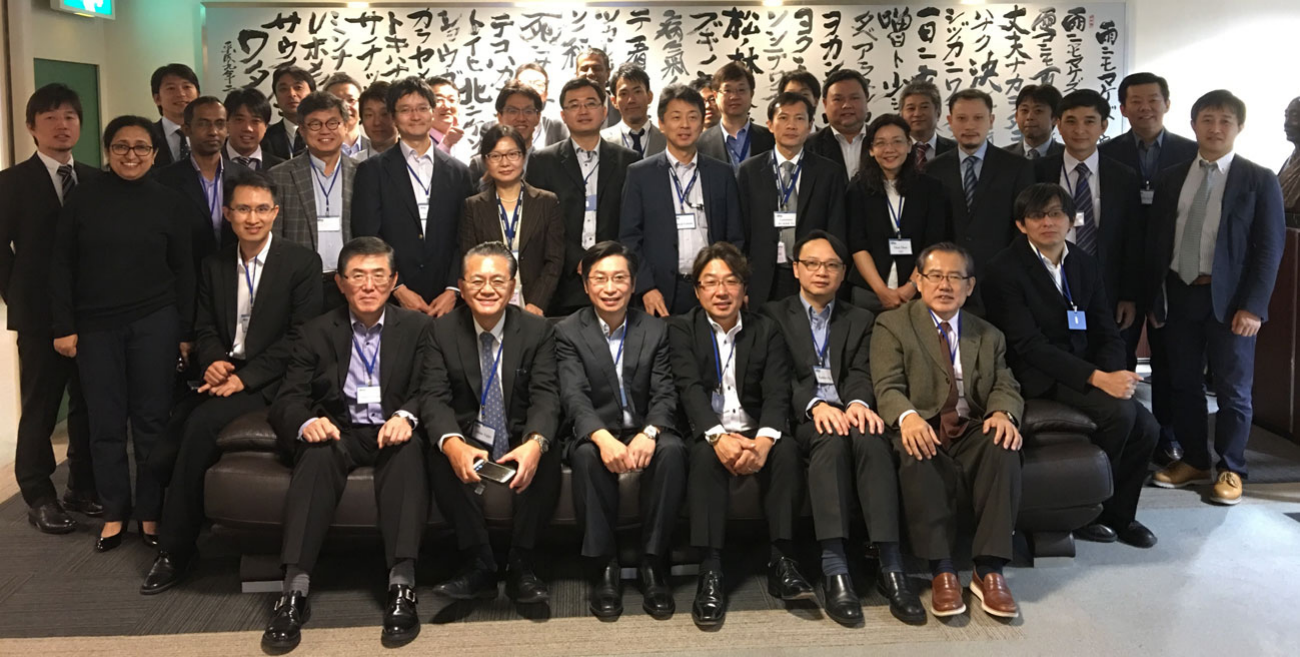
Expert Asian Novel Bio‐Imaging and Intervention Group members at the third consensus meeting.

All of the experts in the panel joined the third consensus face‐to‐face meeting and accomplished the final version of the 10 statements after updating these statements based on the latest evidence. Then, we took an anonymous vote on all of the statements (Fig. 3).

## Results

### Level of agreement for each consensus statement

Agreement on each statement was achieved when more than 80% of the members indicated that they accepted the statement completely or accepted it with some reservation in an anonymous vote using the Delphi method in Sano Hospital. Of the total 10 statements, all statements reached consensus after the first‐round vote (accepted by 100%; Table 3). No statements were rejected or went to the second‐round vote.

**Table 3:** List of statements

	Statements	Evidence	Recommendation	Agreement (%)
1	Adequate bowel preparation is essential for high‐quality colonoscopy	II‐2	B	96
2	Use of antispasmodic agents during colonoscopy is useful for lesion detection	I	C	92
3‐1	Image‐enhanced endoscopy can improve polyp detection in average‐risk patients	I	B	81
3‐2	Chromoendoscopy improves detection of dysplasia in patients with IBD	III	C	92
4	Adenoma detection rate is an important quality indicator and should be monitored	II‐2	A	100
5	Colonoscopy findings should be well documented	III	C	100
6	Complication rates should be monitored as one of the quality indicators for colonoscopy	II	B	92
7	Cecal intubation rate should be monitored as an important quality indicator	II‐2	A	100
8	Cap‐assisted colonoscopy is recommended as an aid to improve polyp detection	I	B	92
9	Macroscopic classification using indigocarmine spray is recommended for characterization of colorectal lesions	III	C	92
10	Image‐enhanced endoscopy and/or magnifying endoscopy can be used by trained endoscopists for accurate prediction of histology	II‐2	B	91

### Consensus statements

Consensus was reached on a total of 10 statements as follows:

#### Statement 1: Adequate bowel preparation is essential for high‐quality colonoscopy

Level of agreement: (i) accept completely 78%; (ii) accept with some reservation 18%; (iii) accept with major reservation 4%; (iv) reject with reservation 0%; (v) reject completely 0%.
Level of evidence: II‐2.
Level of recommendation: B.
Clinical question: Is adequate bowel preparation essential to ensure high‐quality colonoscopy?

High‐quality colonoscopy reduces the risk of CRC by detecting and removing polyps that could potentially develop into cancers. The adenoma detection rate (ADR) is one of the quality indicators for colonoscopy.^12^, ^13^ As bowel preparation quality affects the visualization of colonic mucosa and polyps, it has a direct impact on the ADR. A few studies have shown that the polyp detection rate in patients with adequate bowel preparation is higher than in patients with inadequate bowel preparation.^14^, ^15^ Furthermore, one meta‐analysis has shown that good bowel preparation is significantly associated with higher ADR.^16^ Two tandem colonoscopy studies confirmed that the number of missed polyps was increased in patients with poor bowel preparation.^17^, ^18^

#### Statement 2: Use of antispasmodic agents during colonoscopy is useful for lesion detection

Level of agreement: (i) accept completely 58%; (ii) accept with some reservation 34%; (iii) accept with major reservation 4%; (iv) reject with reservation 4%; (v) reject completely 0%.
Level of evidence: I.
Level of recommendation: C.
Clinical question: Are antispasmodic agents useful for colonoscopy?

Giving antispasmodic agents during screening and surveillance colonoscopy aims to minimize technical difficulties, reduce patient discomfort and reduce the likelihood of missing neoplastic lesions. Several randomized controlled trials (RCT) have reported technical problems related to the use of hyoscine butylbromide as an antispasmodic agent during colonoscopy.^18^, ^19^, ^20^, ^21^, ^22^, ^23^, ^24^ Some RCT have shown that cecal intubation time is shorter in patients given antispasmodic agents,^19^, ^21^ whereas other RCT have concluded that antispasmodic agents are not beneficial for colonoscope insertion.^20^, ^21^, ^22^, ^23^, ^24^ In terms of patient discomfort, one RCT has indicated that antispasmodic agents are beneficial.^21^ Therefore, giving antispasmodic agents to minimize technical difficulties and patient discomfort is still controversial. Recently, several meta‐analyses on the use of antispasmodic agents during colonoscopy for lesion detection have been published.^25^, ^26^, ^27^, ^28^ None of them concluded that the use of antispasmodic agents improved the detection rate of either adenomas or polyps. However, one RCT showed that hyoscine butylbromide increased the number of polyps identified per patient.^29^ Furthermore, a large‐scale retrospective study of data from the English Bowel Cancer Screening Programme showed that the routine use of hyoscine butylbromide was associated with a 30% higher rate of adenoma detection.^30^ Therefore, the use of antispasmodic agents during colonoscopy may be helpful for detection of lesions.

#### Statement 3‐1: Image‐enhanced endoscopy (IEE) can improve polyp detection in average‐risk patients

Level of agreement: (i) accept completely 42%; (ii) accept with some reservation 39%; (iii) accept with major reservation 19%; (iv) reject with reservation 0%; (v) reject completely 0%.
Level of evidence: I.
Level of recommendation: B.
Clinical question: Does the use of IEE improve the detection of polyps in average‐risk patients?

##### Image‐enhanced endoscopy

Image‐enhanced endoscopy is an endoscopic imaging method used to improve the visualization of small blood vessels and mucosal surface patterns on polyps during endoscopy. Generally, IEE consists of two main types: (i) dye‐based IEE (chromoendoscopy); and (ii) equipment‐based IEE. Equipment‐based IEE is subgrouped into optical IEE [narrow‐band imaging (NBI), blue laser imaging (BLI)] and electronic IEE [i‐scan, Fuji Intelligent Color Enhancement (FICE), autofluorescence imaging (AFI)].^30^, ^31^

Two meta‐analyses in 2014 and 2016 found strong evidence that chromoendoscopy enhances the detection of polyps in the colon and rectum.^33^, ^34^ Chromoendoscopy yielded a significantly higher detection rate of neoplastic polyp [odds ratio (OR) 1.53, 95% confidence interval (CI) 1.31–1.79; 7 trials; 2727 participants]. There were no adverse events related to the use of contrast dye in those studies.

##### Narrow‐band imaging

Although recent meta‐analyses have confirmed that NBI is equally as effective as high‐definition white light (HD WL) colonoscopy for detection of colorectal polyps, NBI may be superior to standard‐definition white light (SD WL) colonoscopy.^34^, ^35^, ^36^, ^37^, ^38^ One meta‐analysis confirmed a prolonged withdrawal time in the NBI group and an improved rate of detection for flat adenomas. Second‐generation NBI (190 series), which provides brighter images than the previous model (180 series), has become available. Second‐generation NBI increased the ADR significantly in comparison with HD WL colonoscopy (48% *vs* 34%; *P* = 0.01) in one RCT.^39^ Detection of polyps and sessile serrated polyps was also increased in another RCT, but the rate of detection of adenoma did not change.^40^ However, one study concluded that second‐generation NBI did not improve polyp detection.^41^

##### i‐Scan and FICE

A meta‐analysis in 2014 showed that neither FICE/i‐scan nor AFI improved the ADR over SD/HD WL colonoscopy.^34^ FICE was confirmed not to improve ADR when compared with WL colonoscopy or NBI (25.3% and 24.5%, *P* = 0.75) in subsequent tandem study RCT.^42^ Conflicting results for i‐scan were obtained from a few studies. One tandem study reported a significantly lower adenoma miss rate with i‐scan in comparison with HD WL colonoscopy,^43^ but another concluded that there were no significant differences.^44^ Moreover, one recent non‐randomized controlled study demonstrated that i‐scan had a significantly higher detection rate for adenoma and advanced adenoma in comparison to HD WL endoscopy.^45^

#### Statement 3‐2: Chromoendoscopy improves detection of dysplasia in patients with irritable bowel disease (IBD)

Level of agreement: (i) accept completely 54%; (ii) accept with some reservation 38%; (iii) accept with major reservation 8%; (iv) reject with reservation 0%; (v) reject completely 0%.
Level of evidence: III.
Level of recommendation: C.
Clinical question: Can IEE improve the detection of dysplasia in patients with IBD?

Two meta‐analyses in 2011 and 2013 confirmed the superiority of capsule endoscopy (CE) with targeted biopsy over SD WL colonoscopy with random biopsy (6% increase in the yield for dysplasia).^46^, ^47^ As a result, many international guidelines now recommend the routine use of pancolonic chromoendoscopy with targeted biopsy for neoplasia surveillance in patients with IBD.^48^, ^49^, ^50^, ^51^, ^52^

##### Narrow‐band imaging

Studies comparing NBI with standard WL colonoscopy have not shown any improvement in the detection of dysplasia.^53^, ^54^, ^55^ When compared to chromoendoscopy, NBI seemed to have lower dysplasia detection but the differences were not statistically significant.^56^, ^57^, ^58^ However, in one recent RCT, the new version of NBI was not inferior to pancolonic chromoendoscopy and had a shorter procedure duration.^59^

##### i‐Scan and FICE

There are no published data available for FICE, i‐scan and BLI in the context of colitis surveillance. Therefore, current evidence does not support the use of equipment‐based IEE for surveillance of dysplasia in IBD.

#### Statement 4: Adenoma detection rate is an important quality indicator and should be monitored

Level of agreement: (i) accept completely 83%; (ii) accept with some reservation 17%; (iii) accept with major reservation 0%; (iv) reject with reservation 0%; (v) reject completely 0%.
Level of evidence: II‐2.
Level of recommendation: A.
Clinical question: Can the ADR be used as a quality indicator of colonoscopy?

Several cohort studies have shown a close association between ADR and subsequent risk of post‐colonoscopy CRC (PCCRC) or colonoscopy interval cancers (Table 4).^5^, ^60^, ^61^, ^62^, ^63^ Results from a Polish screening program were first to demonstrate that the ADR is inversely correlated with the risk of interval CRC.^60^ Baxter *et al*.^61^ showed that a low ADR increases the likelihood of proximal PCCRC. Corley *et al*.^5^ reported that the ADR was inversely associated with not only the incidence of CRC, but also the risk of more advanced CRC and CRC mortality, each 1% increase in ADR decreasing CRC mortality by 5%. The only report available for the FIT‐based screening program in Taiwan showed that ADR, together with the cecal intubation rate and baseline colonoscopy findings, was associated with colonoscopy interval cancers.^62^ The benchmark threshold for ADR varies among different programs as it may be affected by the prevalence of adenoma in the population, type of primary screening test used (FIT or colonoscopy), and biological factors such as gender and age. Traditionally, the proportion of subjects with at least one neoplastic lesion among all subjects that underwent colonoscopy was the standard way to define ADR. However, there is a concern that endoscopists may focus on finding one adenoma only and, once they have done so, their attention may wane knowing that they have already contributed to the ADR. This situation may lead to an increase in missed neoplasia (the so‐called “one and done” phenomenon). Modified ADR metrics, such as APP (adenoma per positive participant), ADR‐plus, polypectomy rate, proximal ADR, AADR (advanced adenoma detection rate), or SSADR (sessile serrated adenoma/polyp detection rate), are now being explored.^64^, ^65^, ^66^, ^67^, ^68^, ^69^ It is also important to know that the ADR in a FIT‐based screening program is expected to be higher than that for colonoscopy‐based screening.^70^

**Table 4:** Association of ADR with the risk of incidence of post‐colonoscopy CRC or interval cancers

Author	Study population	Association of ADR and interval CRC risk
Corley *et al*.^5^	Kaiser Permanente Northern California, 314 872 colonoscopies by 136 endoscopists, 1998–2010	ADR: 0.0735–0.1905: reference 0.1906–0.2385: HR = 0.93 (0.70–1.23) 0.2386–0.2840: HR = 0.85 (0.68–1.06) 0.2841–0.3350: HR = 0.70 (0.54–0.91) 0.3351–0.5251: HR = 0.52 (0.39–0.69)
Kaminski *et al*.^60^	Polish national CRC screening program, 45 026 subjects by 186 endoscopists	ADR: ≥0.20: reference 0.15–0.199: HR = 10.94 (1.37–87.01) 0.11–0.149: HR = 10.75 (1.36–85.06) <0.11: HR = 12.50 (1.51–103.43)
Baxter *et al*.^61^	Ontario Cancer Registry 34 312 individuals diagnosed with CRC, 2000–2005	ADR: proximal CRC/distal CRC <0.1: reference 0.1–0.14:1.11 (0.81–1.53)/0.99 (0.73–1.35) 0.15–0.19: 0.75 (0.54–1.04)/0.78 (0.57–1.06) 0.20–0.24: 0.75 (0.52–1.07)/0.82 (0.58–1.16) 0.25–0.29 0.52 (0.35–0.79)/0.87 (0.61–1.24) >30: 0.61 (0.42–0.89)/0.79 (0.54–1.14)
Chiu *et al*.^62^	Taiwanese Nationwide CRC screening program, 29 969 subjects underwent complete colonoscopy after positive FIT during 2004–2009	ADR (hospital level) >0.3: reference 0.30–0.15: HR = 1.57 (0.94–2.61) <0.15: HR = 3.09 (1.55–6.18)
Cooper *et al*.^63^	Surveillance, Epidemiology, and End Results (SEER) Medicare database 57 839 patients aged 69 years underwent colonoscopy during 1994–2005	Polypectomy rate: 0–0.24: reference 0.24–0.33: OR = 0.84 (0.76–0.93) 0.33–0.43: OR = 0.80 (0.72–0.89) >0.43: OR = 0.70 (0.63–0.78)

ADR, adenoma detection rate; CRC, colorectal cancer; FIT, fecal immunochemical test.

#### Statement 5: Colonoscopy findings should be well documented

Level of agreement: (i) accept completely 95%; (ii) accept with some reservation 5%; (iii) accept with major reservation 0%; (iv) reject with reservation 0%; (v) reject completely 0%.
Level of evidence: III.
Level of recommendation: C.
Clinical question: How should findings of colonoscopy be documented?

For each lesion detected by colonoscopy, location, size, morphology, optical diagnosis and intervention should be documented, and images captured. Documentation of procedural findings is central to the practice of quality colonoscopy. The report is an essential communication tool, serving as a permanent clinical and legal record of the procedure, and facilitating exchange of information between health‐care providers.^71^ Quality of colonoscopy is under scrutiny,^10^, ^12^, ^72^ and performance indicators are known to predict the incidence of post‐colonoscopy (interval) CRC.^5^, ^10^ Multiple studies have shown deficits and variations in the quality of reporting. Reports are often incomplete, with lack of documentation of key procedural elements such as bowel preparation quality, polyp size and morphology.^43^, ^74^, ^75^ As in other aspects of colonoscopy practice, the quality of colonoscopy reporting varies significantly between endoscopists, endoscopy units, and geographical regions.^76^, ^77^, ^78^ Electronic reporting systems are known to be associated with more complete reporting.^79^, ^80^ The European Society of Gastrointestinal Endoscopy (ESGE) position statement on reporting systems in endoscopy has emphasized the need for structured terminology with limitations to the use of free‐text data entry.^81^ This requires a standardized language for GI endoscopy and the use of “minimal standard terminology” for recommended reporting terms and structure.^82^, ^83^ In the USA, the Quality Assurance Task Group of the National Colorectal Cancer Round Table (NCCRT) has developed a standardized colonoscopy reporting and data system (CO‐RADS) to improve the quality of colonoscopy.^79^ The recommendations for colonoscopic findings include standardized descriptors for colonic polyps, emphasizing that communication of findings is an important determinant of risk status and subsequent surveillance. For each polyp, CO‐RADS‐recommended descriptors are anatomical location, size, morphology, method of removal, completeness of removal, retrieval and submission for pathological evaluation. Documentation of lesion location, size, morphology, optical diagnosis and intervention for each lesion at colonoscopy allows determination of the risk for metachronous neoplasia and the need for subsequent clinical follow up and colonoscopic surveillance.^84^, ^85^ These findings also facilitate reporting of continuous quality improvement targets such as the ADR.^14^ Without adequate documentation, continuous quality improvement processes are not possible and opportunities for improvement cannot be identified.

#### Statement 6: Complication rates should be monitored as one of the quality indicators for colonoscopy

Level of agreement: (i) accept completely 50%; (ii) accept with some reservation 42%; (iii) accept with major reservation 8%; (iv) reject with reservation 0%; (v) reject completely 0%.
Level of evidence: II.
Level of recommendation: B.
Clinical question: Should complication rates be monitored as an indicator of colonoscopy quality?

As the indications for endoscopy increase, particularly in relation to therapeutic procedures, clinicians will have to deal with an increasing number of complications. Early diagnosis and interventions are important for management of complications related to endoscopy, and treatment sometimes requires a multidisciplinary approach.^86^ Complications vary from simple abdominal pain to more serious complications such as bleeding and perforation.^87^, ^88^, ^89^ There are legal implications to these events, which may be unfavorable for physicians even if there is no clear correlation between the medical action and a worsening health condition.^90^ Establishing a good doctor–patient relationship before any endoscopic procedure with realistic expectations can alleviate some of these stresses and help to provide improved clinical care when adverse events occur.^91^, ^92^ Monitoring of complications has already become a standard quality indicator in many countries, and bleeding rate (<1%) and perforation rate (<0.1%) are often defined as the minimum threshold for assuring quality of endoscopic procedures.^93^

#### Statement 7: Cecal intubation rate should be monitored as an important quality indicator

Level of agreement: (i) accept completely 89%; (ii) accept with some reservation 11%; (iii) accept with major reservation 0%; (iv) reject with reservation 0%; (v) reject completely 0%.
Level of evidence: II‐2.
Level of recommendation: A.
Clinical question: Does a high cecal intubation rate improve the detection rate of adenomas and serrated lesions and decrease the incidence of interval cancers?

In terms of definition, cecal intubation is achieved when the tip of the colonoscope is passed beyond the ileocecal valve into the cecum.^94^ As interval or missed cancers and missed precancerous lesions are likely to occur in the right colon, ensuring intubation into the cecum is important.^95^, ^96^, ^97^, ^98^, ^99^, ^100^ In 2002, the cecal intubation rate was recommended by the US Multi‐Society Task Force (MSTF) on CRC as an indicator of colonoscopy quality. Cases with colon obstructed by CRC were generally included in the calculation of cecal intubation rates.^94^ In recent years, studies have been conducted to survey the impact of the cecal intubation rate on colorectal neoplasia detection. In the Canadian CRC screening cohort and the English Bowel Cancer Screening Programme, the cecal intubation rate was positively associated with the detection rate of adenomas and serrated lesions.^26^, ^101^ A population‐based analysis of CRC patients has shown that they were more likely to undergo incomplete colonoscopy.^99^ A study comparing the risk of interval CRC among endoscopists who used different cecal incubation rates found that interval cancers were less common among those with higher rates (>90%) relative to the others (<80%).^61^ Jover *et al*.^102^ showed that a higher cecal intubation rate was associated with detection of proximal adenomas, and also with a higher ADR. Therefore, the cecal intubation rate should be monitored as an important quality indicator. To ensure the quality of colonoscopy, the MSTF has recommended that the cecal intubation rate should be more than 90% in all cases, and 95% in screening cases. Documentation of cecal intubation must be included in all endoscopic reports with photography of visualized landmarks when available.^103^

#### Statement 8: Cap‐assisted colonoscopy is recommended as an aid to improve polyp detection

Level of agreement: (i) accept completely 42%; (ii) accept with some reservation 50%; (iii) accept with major reservation 8%; (iv) reject with reservation 0%; (v) reject completely 0%.
Level of evidence: I.
Level of recommendation: B.
Clinical question: Does CAC improve polyp detection?

Cap‐assisted colonoscopy is universally available in Asian countries and can marginally improve polyp or adenoma detection. However, it may only be beneficial for selected targets, such as trainees and endoscopists with a longer withdrawal time. Removal of all neoplastic lesions by colonoscopy can reduce the incidence of CRC and associated mortality and, hence, high‐quality examination is crucial for effective CRC prevention. The ADR is an important quality indicator associated with the incidence of interval cancer or post‐colonoscopy CRC. Considering the medical and economic situation in Asia, CAC is the only affordable device available for improving the ADR. Although the results from several studies have been conflicting,^104^, ^105^, ^106^, ^107^, ^108^, ^109^ many have reported that CAC improved the ADR.^110^, ^111^, ^112^, ^113^, ^114^, ^115^, ^116^, ^117^, ^118^ Furthermore, three meta‐analyses have demonstrated a marginal benefit for polyp or adenoma detection,^119^, ^120^, ^121^ and one other study has also reported the usefulness of CAC for detection of serrated polyps.^122^ Although some authors have suggested that CAC may be beneficial for selected endoscopists only (e.g. trainees with a longer withdrawal time, and with a low ADR),^109^, ^118^ further studies to clarify this problem are desirable. Although CAC may shorten the cecal intubation time^104^, ^105^, ^107^, ^109^, ^113^, ^117^ and reduce the level of patient discomfort,^104^, ^105^, ^107^ it may make rectal retroflection difficult.

#### Statement 9: Macroscopic classification using indigocarmine spray is recommended for characterization of colorectal lesions

Level of agreement: (i) accept completely 63%; (ii) accept with some reservation 29%; (iii) accept with major reservation 8%; (iv) reject with reservation 0%; (v) reject completely 0%.
Level of evidence: III.
Level of recommendation: C.
Clinical question: Can chromoendoscopy improve the characterization of colorectal lesions?

The Paris classification is a globally used macroscopic classification that divides superficial neoplastic lesions into polypoid (Ip, Is) and non‐polypoid (IIa, IIb, IIc, III).^123^ Recognition of gross appearance and macroscopic classification assists the diagnosis of neoplastic or non‐neoplastic lesions and prediction of submucosal invasion.

**Figure 4:**
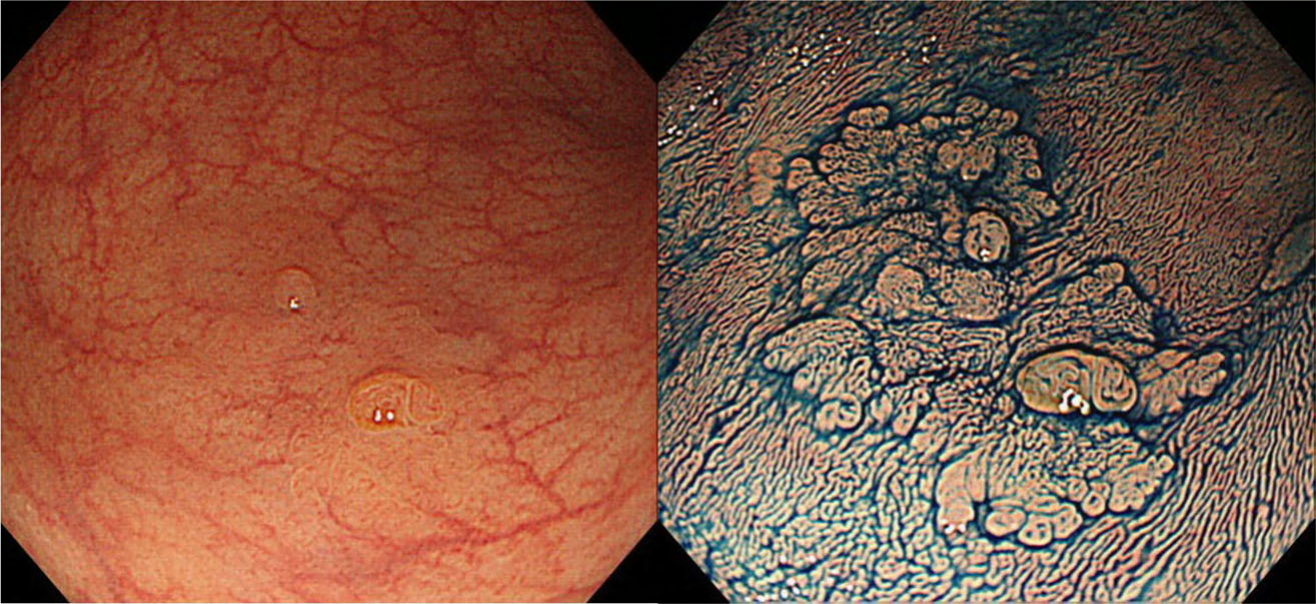
Case of sessile serrated polyp (SSP) with cytological dysplasia in the sigmoid colon shows the importance of chromoendoscopy. (Left) Only two elevated, polypoid areas are clearly observable with conventional white light imaging. (Right) Macroscopic characterization (IIa) is markedly clear after indigocarmine spraying.

The incidence of submucosal invasion is markedly higher for the non‐polypoid type relative to the polypoid type (42.4% *vs* 21.0%) in the National Colorectal Cancer Screening in Japan.^124^ The alternative morphological term “laterally spreading tumor” (LST), proposed by Kudo, is now widely accepted in both Asian and Western countries.^125^ Although this is not an official term, the two LST subtypes – granular type (LST‐G) and non‐granular type (LST‐NG) – are significantly associated with frequency of submucosal invasion (LST‐NG 14% *vs* LST‐G 7%; *P* < 0.01).^126^, ^127^, ^128^ However, the macroscopic type of some lesions is difficult to recognize (Fig. 4). For such cases, contrast chromoendoscopy using, for example, indigocarmine dye spraying provides additional information that can improve macroscopic characterization. Furthermore, staining chromoendoscopy using crystal violet or methylene blue with magnifying endoscopy is useful for detailed observation of the pit pattern (surface pattern).

#### Statement 10: IEE and/or magnifying endoscopy can be used by trained endoscopists for accurate prediction of histology

Level of agreement: (i) accept completely 70%; (ii) accept with some reservation 21%; (iii) accept with major reservation 9%; (iv) reject with reservation 0%; (v) reject completely 0%.
Level of evidence: II‐2.
Level of recommendation: B.
Clinical question: Can trained endoscopists predict histology accurately using IEE and/or magnifying endoscopy?

Image‐enhanced endoscopy is a method for enhancing the visualization of small blood vessels and minute patterns on the mucosal surface and can include NBI, FICE/BLI, i‐scan, confocal laser endomicroscopy (CLE), AFI, or chromoendoscopy.^32^ A number of articles have assessed the diagnostic performance of IEE with or without magnification for differentiating non‐neoplastic from neoplastic polyps, and the overall diagnostic accuracy achieved is mostly more than 90%.^129^, ^130^, ^131^, ^132^, ^133^, ^134^, ^135^, ^136^, ^137^, ^138^, ^139^, ^140^, ^141^ Meta‐analyses of NBI, i‐scan, FICE, AFI, or CLE have shown that all techniques except for AFI achieved satisfactory diagnostic performance.^138^, ^139^, ^140^, ^141^, ^142^, ^143^ However, the performance levels for histological prediction by non‐experts are not as good as those by experts (e.g. routine use of NBI for more than 5 years).^148^, ^149^ Some studies have shown that training modules or continuous feedback *in vivo* are beneficial for non‐experts in improving their diagnostic performance.^145^, ^146^, ^147^ For diagnosis of CRC invasion depth, magnifying chromoendoscopy yielded the highest accuracy at 98.8%.^152^ It has also been reported that NBI and FICE/BLI could be useful for determining therapeutic strategies including endoscopic resection or surgery.^153^, ^154^, ^155^, ^156^, ^157^, ^158^, ^159^, ^160^, ^161^

## Discussion

**Figure 5:**
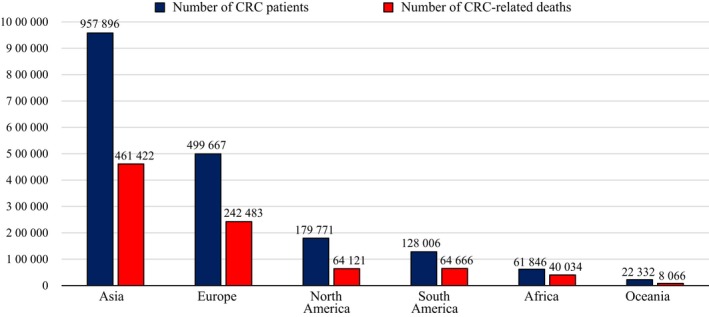
Estimated number of colorectal cancer (CRC) patients and CRC‐related deaths in 2018.

Colorectal cancer is one of the leading malignant diseases in Asia. In comparison with other continents, total number of CRC patients in the Asian population is markedly high according to the International Agency for Research on Cancer (IARC). In 2018, the Global Cancer Observatory (GLOBOCAN) estimation project showed that the total number of CRC patients and CRC‐related deaths in Asia was estimated to be approximately 1 million and 500 000, respectively (Fig. 5). Although these gross numbers are affected by the explosive growth of the Asian population, it remains an important problem in Asia that the number of people suffering from CRC is the highest among all continents in the world.^1^ It has been clarified that CRC‐related deaths can be prevented by identification and removal of early‐stage neoplasia.^4^ Nevertheless, the detection rate and diagnostic accuracy for early‐stage colorectal neoplasia vary among Asian countries. Quality of diagnostic colonoscopy must be improved or managed appropriately in each country to avoid missed cancers, yet no consensus for diagnostic colonoscopy of colorectal neoplasia has emerged in Asia.

Against this background, we have developed an Asian expert‐based consensus on fundamental knowledge of colonoscopy to minimize differences in the quality of diagnostic colonoscopy throughout Asia. This is the first Asian consensus to be formulated for diagnostic colonoscopy based on scientific evidence and opinions from thorough discussions by Asian experts. This study achieved consensuses on several important points: adequate bowel preparation, use of antispasmodic agents, ADR using IEE or CAC, documentation methods, complication rates, cecal intubation rate, precise recognition of macroscopic classification, and histological prediction using IEE and/or magnifying endoscopy.

Although several consensus statements regarding screening, surveillance, and quality assurance have previously been published by different societies, consensuses on diagnostic endoscopy are limited.^1^, ^3^, ^4^, ^11^, ^84^, ^162^, ^163^, ^164^, ^165^, ^166^Our current Asian consensus differs in that we have focused on essential knowledge for achieving high performance in detection and diagnosis of colorectal neoplasia. To meet the urgent demand of standardized diagnostic strategy especially in Asia, our consensus was designed and developed to be simple and widely acceptable to many countries. In contrast to clinical guidelines, the consensus statement has an advantage in that it is free from cultural, economic, ethical, political, and technical concerns. For this reason, Asian countries may not accept or adopt entire Western guidelines for colonoscopy such as ESGE guidelines and American Society for Gastrointestinal Endoscopy guidelines.^9^, ^84^, ^85^, ^167^ Furthermore, reviewing the literature alone cannot always provide sufficient evidence to answer certain clinical questions on diagnostic colonoscopy; therefore, expert opinions through the Delphi method can guide Asian endoscopists to standardize endoscopic diagnosis.

This consensus will provide a basis for further elaboration and modification to suit the needs of individual Asian countries in the context of current endoscopic diagnosis of early‐stage colorectal neoplasia. We expect that this consensus will contribute to standardization of the quality of diagnostic colonoscopy in Asia and, hopefully, in other parts of the world. Future perspective of our group is to develop consensus statements on endoscopic treatment of GI neoplasia.^168^

## Conclusion

The Asian expert members have held meetings to refine various points of consensus related to diagnostic endoscopy and have developed an expert‐based consensus on standards of diagnostic colonoscopy for early‐stage neoplasia.

## Conflicts of Interest

The consensus meeting was supported by a non‐government organization named Asian Novel Bio‐Imaging and Intervention Group (ANBI^2^G) which focused on training and education of endoscopic diagnosis and management of early gastrointestinal cancers in Asia. The ANBI^2^G was supported by donations from Olympus Corporation and Takeda Pharmaceutical Company Limited. The authors have no conflicts of interest to declare in relation to this article.
